# Psychometric Properties of the Recovery Assessment Scale (RAS) in Spain: Confirmatory Factor Analysis and Metric Superiority of the 18-Item Version (RAS-18)

**DOI:** 10.62641/aep.2404

**Published:** 2026-10-15

**Authors:** María Isabel Ruiz-Granados, Javier Herruzo-Cabrera, José Antonio Garrido Cervera, Antonio José Sánchez-Guarnido

**Affiliations:** ^1^Department of Psychology, University of Córdoba, 14071 Córdoba, Spain; ^2^Clinimetrics in Physiotherapy Group (CTS 631), Department of Physiotherapy, Faculty of Health Sciences, University of Malaga, 29071 Malaga, Spain; ^3^Clinimetrics Group (F-14), Malaga Biomedical Research Institute and Nanomedicine Platform (IBIMA Bionand Platform), 29590 Malaga, Spain; ^4^Department of Physiotherapy, University of Malaga, 29071 Malaga, Spain; ^5^Mental Health, Antequera Hospital, 29200 Malaga, Spain; ^6^Mental Health, Virgen de las Nieves University Hospital, 18014 Granada, Spain

**Keywords:** personal recovery, RAS-18, psychometrics, confirmatory factor analysis, severe mental disorder

## Abstract

**Background:**

The adoption of the recovery model in mental health creates a need for validated instruments that go beyond the assessment of clinical symptoms. The Recovery Assessment Scale (RAS) is the most widely used test internationally. However, the need to balance the validity of psychological measurement with reduced administration time has led to the search for shorter versions. In this vein, there is an 18-item version that has not yet been validated in Spain. To study the psychometric properties of the RAS in Spain, analysing the 41-, 24- and 18-item versions, to determine the version with the best fit and greatest parsimony.

**Methods:**

The questionnaires were administered to a sample of 307 patients with severe mental disorders (SMDs) treated in public mental health services, and a confirmatory factor analysis (CFA) was performed testing the three versions. Concurrent and divergent validity were examined using the Individual Recovery Outcomes Counter (I.ROC) scale and the Health of the Nation Outcome Scales (HoNOS) scale, respectively. A retest was also performed after 15 days to check temporal stability.

**Results:**

The RAS-18 demonstrated superior metrics and greater parsimony (with improvements in chi-square, Comparative Fit Index (CFI), Root Mean Square Error of Approximation (RMSEA), Akaike Information Criterion (AIC) and Bayesian Information Criterion (BIC)), whilst maintaining good results in internal consistency (alpha = 0.892; ω = 0.901), test-retest reliability (intraclass correlation coefficient (ICC) = 0.864) and convergent validity with the I.ROC (r = 0.784).

**Conclusions:**

The study recommends the use of the RAS-18 in Spain in clinical and research contexts. This version improves the assessment of personal recovery, reducing administration time without sacrificing the psychometric accuracy of the construct.

## Introduction

Mental health care has undergone a paradigm shift in recent decades, moving from a traditional biomedical model, focused almost exclusively on pathology, deficit and symptomatic remission (clinical recovery), to the so-called “personal recovery model” [1]. While clinical recovery is understood as the absence of symptoms and is usually assessed by a professional, personal recovery is a more subjective process. This approach, supported by theoretical frameworks such as CHIME (Connection, Hope, Identity, Meaning and Empowerment) [2] and the stages model of Andresen *et al*. [3], conceptualises health not as the absence of illness, but as the ability of people with Severe Mental Illness (SMI) to build a life with purpose, meaning and active social roles, regardless of the persistence of certain symptoms.

In the Spanish context, this change in perspective is not merely theoretical, but has begun to permeate the National Health System’s mental health regulations and strategies, placing the focus on autonomy, human rights and full citizenship for patients [4, 5]. However, the implementation of this model in clinical practice faces the challenge of operationalising and measuring a construct that is, by definition, intrinsically subjective. Classic psychometric tools, designed to measure the intensity of psychotic or affective symptoms, are insufficient and inadequate for capturing salutogenic dimensions such as hope, empowerment, or sense of purpose in life [6, 7].

To move towards evidence-based practice that respects this paradigm, it is necessary to have psychometrically robust Patient-Reported Outcome Measures (PROMs) that are. The Recovery Assessment Scale (RAS), initially developed by Giffort *et al*. [8] and whose theoretical basis was consolidated by Corrigan *et al*. [9], is considered the international “gold standard” in this field. Recent reviews [10, 11] continue to identify it as the most widely used and cited measure of recovery in the global scientific literature, highlighting its ability to cover key domains of the CHIME framework.

Regarding its internal structure, the psychometric literature has pointed out significant problems in the original 41-item version (RAS-41). Several international studies, including validations in Japan [12], Australia [13] and Argentina [14], have warned that the RAS-41 does not offer good results in factor analyses, showing instability, semantic redundancies and high residual correlations that compromise construct validity. The excessive length of the instrument not only increases the cognitive load for the person being assessed, but also makes it difficult to obtain a clean and replicable factor structure.

Given these metric limitations, the reduced 24-item version (RAS-24) proposed by Corrigan *et al*. [15] was consolidated, establishing a five-factor structure that has become the benchmark: (1) personal confidence and hope, (2) willingness to ask for help, (3) goal and success orientation, (4) trust in others, and (5) non-domination by symptoms. This five-factor structure has proven to be theoretically sound, and Saavedra *et al*. [16] recently validated it in Spain with good results. However, the question arises as to whether this five-dimensional structure is maintained or even better defined in even shorter versions. In this regard, recent work such as the Chilean validation by Caqueo-Urízar *et al*. [17] stands out, comparing versions of 24, 21, and 18 items. Their findings confirmed that the original five-factor structure of the RAS-24 fits perfectly with the refined 18-item version (RAS-18). Furthermore, they observed that the RAS-18 performed better in terms of absolute and comparative fit indices than the RAS-24, suggesting that the elimination of redundant items does not alter the theoretical structure but rather clarifies it.

In Spain, although we have validation of the RAS-24, we lack studies that compare, in the same clinical sample, whether the reduction to 18 items represents a loss of relevant information or whether, on the contrary, it refines the measure by eliminating statistical “noise”. This study aims to fill this gap by analysing the psychometric properties of the RAS in the Spanish population. To this end, we will check whether the problems found in previous studies in the RAS-41, and we will compare the psychometric properties of the RAS-24 and RAS-18. Our working hypothesis is that, following the principle of scientific parsimony, the 18-item version will offer superior psychometric fit, facilitating its routine use without reducing reliability or validity.

## Methods

### Design and Participants

An observational study with a cross-sectional instrumental design was conducted, complemented by a longitudinal phase of repeated measures to assess temporal stability (test-retest). The methodological development and data collection were carried out between January and December 2023.

The final sample consisted of 307 participants diagnosed with Severe Mental Disorder (SMD), recruited through consecutive non-probabilistic sampling in various facilities of the Andalusian public mental health network (Community Mental Health Units, Day Hospitals, Therapeutic Mental Health Communities, and Rehabilitation Units). Inclusion criteria stipulated: (1) age between 18 and 65 years; (2) primary clinical diagnosis of SMI according to the International Classification of Diseases (ICD-10), [18] corroborated by a structured clinical interview by the referring physicians. Within the diagnostic categories of SMI, the following were included: schizophrenia and other psychotic disorders (ICD-10 F20-F29), severe affective disorders including bipolar disorder and recurrent depressive disorder (ICD-10 F30-F39), and severe personality disorders (ICD-10 F60-F69) [19] preserved cognitive capacity to read and understand the scales in Spanish. The following were established as exclusion criteria: (1) presence of severe cognitive impairment or an episode of acute psychomotor agitation at the time of evaluation; (2) active substance intoxication.

The final sample size met the statistical power criteria recommended for confirmatory factor analysis, exceeding the traditional ratio of 10:1 subjects per item for short versions and the threshold of 200 cases necessary to ensure the stability of maximum likelihood estimates [20, 21, 22, 23].

### Instruments

RAS-41: The original complete version of 41 items was applied. For the purposes of the comparative analysis of the study, the items corresponding to the reduced versions of 24 [15] and 18 items were extracted and analysed as nested subsets within the overall data matrix. This extraction was performed respecting the exact matching of the items enumerated and validated in the cited reference studies (Corrigan *et al*. [15] for the RAS-24 and Caqueo-Urízar *et al*. [17] for the RAS-18), thus ensuring that the fit comparisons were made on the same sample basis.

Individual Recovery Outcomes Counter (I.ROC): This scale was used as a reference criterion for convergent validity. The I.ROC is a 12-item tool designed specifically to assess personal recovery outcomes. It has been validated internationally and in Spain [24].

Health of the Nation Outcome Scales (HoNOS): Used to examine divergent validity. This hetero-administered scale (completed by the clinician) assesses symptom severity, social functioning, and behavioral problems [25]. The inclusion of this test will allow us to contrast subjective recovery (assessed by the patient) with objective clinical status (assessed by the professional).

### Procedure

The study complied at all times with the principles of the Declaration of Helsinki. The research protocol was approved by the Córdoba Research Ethics Committee in Andalusia. All participants were informed verbally and in writing about the objectives of the study and signed an informed consent form before being included.

The adaptation of the RAS for use in Spain was based on a rigorous translation and cultural adaptation procedure. This process included a double direct translation, an independent back-translation to verify conceptual equivalence, and a final review by a committee of experts (researchers and clinicians) to ensure the comprehension and suitability of the instrument in the Spanish clinical population.

Data collection was carried out through a clinical interview and assisted self-administration. The staff in charge of administration, who were unaware of the study’s objectives and hypotheses (blind administration), ensured that the participants correctly understood the items. To assess the temporal stability of the results, a random subsample of 103 participants was re-evaluated. This second evaluation was carried out after an interval of 15 days, a period considered optimal for minimising both memory effects and the possibility of significant clinical changes.

### Data Analysis

Descriptive statistics were used for the analysis of the sample. Subsequently, reliability was assessed using internal consistency (Cronbach’s alpha and McDonald’s omega) and temporal stability (using the intraclass correlation coefficient (ICC)). External construct validity was analysed using Pearson correlations with reference measures (I.ROC and HoNOS). These analyses were performed using the SPSS v26 statistical package (IBM, Corp,. Armonk, New York, USA).

For structural validity, the R environment (R Foundation for Statistical Computing, Vienna, Austria) was used, employing the lavaan package. Given that the RAS items are ordinal in nature and the data showed significant deviations from multivariate normality (significant Mardia coefficient), the Maximum Likelihood Robust (MLR) estimator was used. This method provides corrected standard errors and chi-square statistics (Satorra-Bentler) that are insensitive to the lack of normal.

Three models were specified and compared:

Model 1 (RAS-41): Complete original structure.

Model 2 (RAS-24): Reduced 5-factor structure proposed by Corrigan *et al*. [15].

Model 3 (RAS-18): Refined 5-factor structure proposed by Caqueo-Urízar *et al*. [17].

The goodness of fit of the models was assessed using a combination of absolute and incremental indices (Satorra–Bentler chi-square (SB-χ^2^), Comparative Fit Index (CFI), Tucker–Lewis Index (TLI), Root Mean Square Error of Approximation (RMSEA)). For model comparison, priority was given to the Akaike Information Criterion (AIC) and Bayesian Information Criterion (BIC), where lower values indicate a better balance between model fit and complexity.

## Results

### Sample Characteristics

The sociodemographic and clinical data of the sample can be seen in Table 1. The mean age of the participants was 40.46 years (standard deviation (SD) = 10.50), with a range of 18 to 65 years and a mean of 15.6 years (SD = 8.68) of evolution since the first diagnosis. Regarding the diagnosis, schizophrenia spectrum disorders and other psychotic disorders predominated (74.9%), followed by personality disorders (14.0%) and severe affective disorders (11.1%).

**Table 1.** **Demographic characteristics of the sample (n = 307)**.

Base characteristics		n	%
Gender			
	Female	124	40.4
	Male	183	59.6
Marital status			
	Single	239	77.8
	Married/Partner	24	7.8
	Divorced/Separated	42	13.7
	Widowed	2	0.7
Educational level			
	No studies	18	5.9
	Primary (EGB-ESO)	143	46.6
	Secondary (BUP-bachiller-vocational)	106	34.5
	University degree/Graduate	40	13.0
Employment			
	Unemployed	92	30.0
	Working	19	6.2
	Retired/Pensioner	182	59.3
	Student	13	4.2
	Volunteer	1	0.3
Distribution by diagnoses			
	Schizophrenia and other psychotic disorders (F20-F29)	230	74.9
	Severe affective disorders (F30-F39)	34	11.1
	Personality disorders (F60-F69)	43	14.0

EGB, General Basic Education; ESO, Compulsory Secondary Education; BUP, Unified Multipurpose Baccalaureate.

### Internal Structure: Comparison of Models

The RAS-41 model presented serious statistical problems, failing to converge to a stable result and exhibiting a non-positive definite variance-covariance matrix. These issues indicate a high degree of interdependence (severe multicollinearity) and redundant information (redundancy) amongst the 41 items. These failures are attributable to the existence of items with almost identical or semantically overlapping phrasing, which generates severe collinearity, rendering it unsuitable for structural analysis and making its use inadvisable.

When comparing the short versions (see Table 2), the RAS-18 model exhibited a more favourable fit compared to the RAS-24. The χ^2^ statistic was reduced by almost half (319.6 vs 577.6), indicating a much smaller discrepancy with the empirical data. The incremental (CFI = 0.885 vs 0.849) and absolute (SRMR = 0.082 vs 0.079) fit indices also improved. Finally, both the AIC (89.3 vs 110.8) and the BIC (248.2 vs 308.7) improved.

**Table 2.** **Model fit indices**.

Model	χ ^2^	CFI	TLI	RMSEA	AIC	BIC
Model (RAS-24)	577.6	0.849	0.828	0.079	110.8	308.7
Model (RAS-18)	319.6	0.885	0.860	0.082	89.3	248.2

RAS, Recovery Assessment Scale; CFI, Comparative Fit Index; TLI, Tucker–Lewis Index; RMSEA, Root Mean Square Error of Approximation; AIC, Akaike Information Criterion; BIC, Bayesian Information Criterion.

### Factor Structure of the RAS-18 Model

Having established the superiority of the 18-item version, its internal parameters were analysed (see Fig. 1). All standardised factor loadings were statistically significant (*p* < 0.001). No weak loadings (<0.40) were observed, and the majority were above 0.60. Only within Factor 4 (Trust in others) did item 40 show a lower loading (λ = 0.42). However, it was decided to retain it due to its theoretical relevance and because it exceeds the frequently accepted minimum threshold (>0.40). The confirmed factor structure retains the five original latent variables, which are moderately to highly correlated with one another, supporting the theoretical coherence of the instrument: (1) Personal Confidence and Hope, (2) Willingness to Ask for Help, (3) Hopeful Goals Orientation, (4) Confidence in Others, and (5) Symptoms Non-Dominance.

**Fig. 1.**
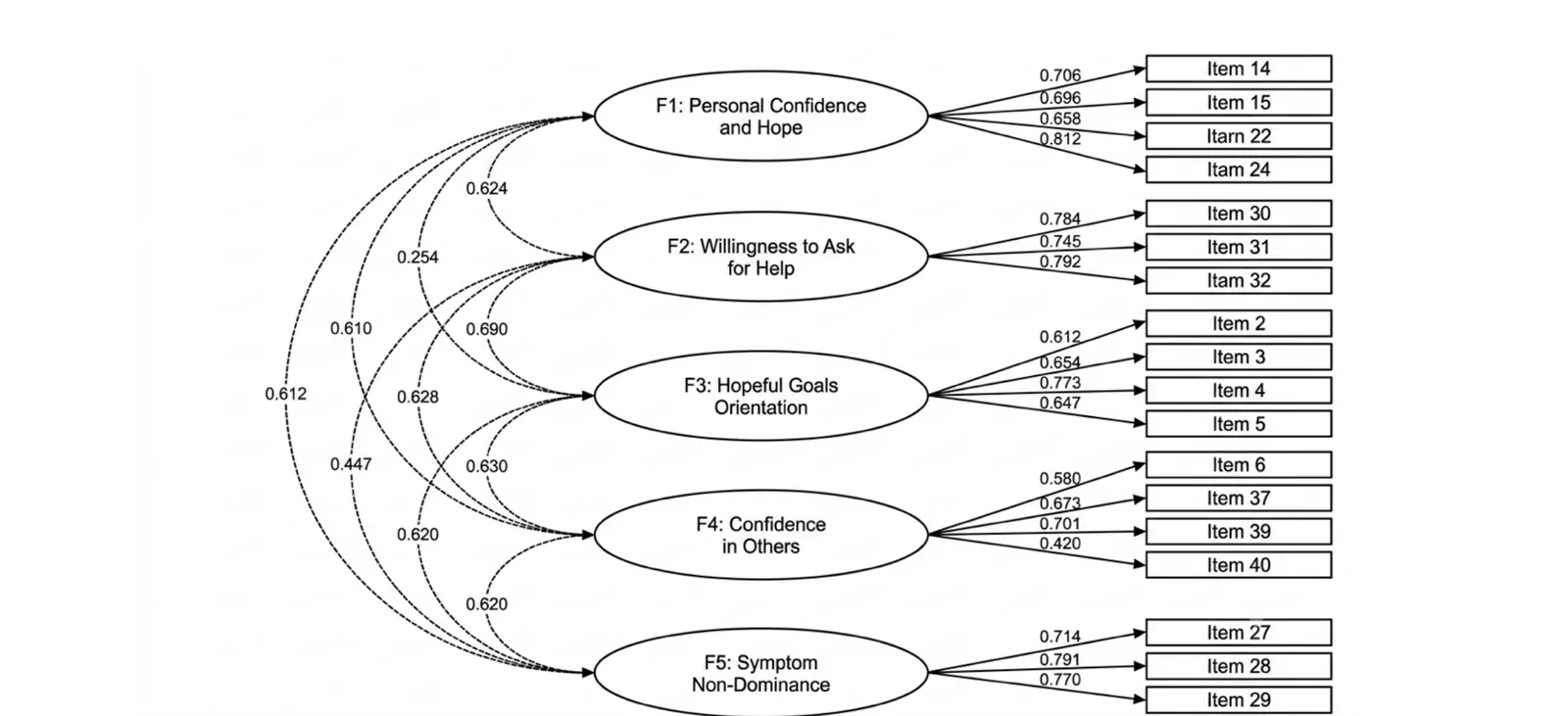
**Final confirmatory factor analysis model**.

### Reliability and Stability

The RAS-18 version obtained a Cronbach’s alpha of 0.892 and a McDonald’s omega of 0.901. With regard to test-retest reliability, the analysis showed an ICC of 0.864. This value indicates good reliability, ensuring that scores are reproducible in the absence of actual clinical changes.

### Convergent and Divergent Validity

The external validity analysis supported the theoretical fit of the instrument with the recovery model. Regarding convergent validity, a strong positive correlation was found with the I.ROC (r = 0.784, *p* < 0.01). In contrast, for divergent validity, the relationship with clinical severity assessed objectively by the clinician (HoNOS) was inverse and of lesser magnitude (r = –0.442, *p* < 0.01).

## Discussion

The findings of this study are conclusive and have direct implications for both theory and healthcare practice: the RAS-41 is psychometrically ineffective due to structural redundancies, while the RAS-18 emerges as the superior option in terms of efficiency, goodness of fit, and validity. Although the CFI index obtained for the RAS-18 (0.885) is slightly below the 0.90 threshold traditionally considered excellent [17], this value is methodologically acceptable and expected in structural models that evaluate highly subjective, broad, and multifaceted psychosocial constructs such as “personal recovery”. The empirical superiority demonstrated over the previous 24-item model justifies its robustness.

Our findings not only confirm what was already known, but also expand on it: in psychometrics, it does not always take much to measure well. In fact, by using the 18-item version, we have gained in efficiency without sacrificing measurement quality. While Corrigan *et al*. [15] already warned about statistical noise in the original version, our data suggest that the RAS-24—considered the standard until now—still retains some inefficiencies. The difference observed in the information criteria (AIC and BIC) in favour of the RAS-18 reflects that we have managed to eliminate items that probably contributed more error variance or diffuse cross-loadings than substantive information about the construct. By refining the scale, a clearer factorial structure emerges that better represents the latent dimensions of recovery. Furthermore, the results obtained for the 18-item model in terms of internal consistency, test-retest reliability, and concurrent and divergent validity are highly satisfactory, clearly validating this version for use in Spain.

From a pragmatic perspective, the validation of the RAS-18 is particularly timely for the National Health System. In public health settings characterised by high care pressure and limited consultation times, reducing administration time is a critical factor for viability [10]. Having a tool that can be completed in just 3–5 minutes reduces patient fatigue, minimises the rate of missing data, and greatly facilitates its incorporation into routine assessment protocols. This allows clinicians to monitor recovery progress, something that is unfeasible with 41-item batteries, and facilitates its potential integration into mental health apps and telecare platforms, where brevity is an essential requirement for user engagement [26, 27].

The study also has other strengths, notably the rigorous procedure for translating and adapting the I.ROC into Spanish. This process, which included independent back-translation, guarantees the quality of the measurement. The choice of the I.ROC as a contrasting tool stands out, whose recent independent validation in a Spanish population in a previously published study [19] guarantees the solidity of the concurrent validity analysis. Another key strength is the sample of 307 participants with SMD, considered adequate to ensure the robustness of the statistical analyses. Furthermore, the results obtained are consistent with the findings of research carried out in other international contexts.

Despite the robustness of the analyses, the study has some limitations. Thus, the incidental sampling carried out in a single autonomous community requires caution when generalising the data. Another methodological limitation to consider is the context effect or “nested effect”: since the response to the 18 items was statistically extracted from the complete 41-item questionnaire, it is possible that the results are influenced by fatigue or psychological priming derived from the preceding items. It would be beneficial for future research to administer the RAS-18 in isolation and independently. Furthermore, in relation to future research, it would be beneficial to design prospective longitudinal studies to evaluate the sensitivity to change of the RAS-18 after specific psychosocial interventions aimed at recovery, which would reinforce its usefulness as an indicator of therapeutic outcome.

## Conclusions

The Spanish adaptation of the RAS-18 is a psychometrically robust instrument, surpassing its more extensive 24- and 41-item predecessors in terms of parsimony and fit. Its ability to effectively discriminate between personal recovery and clinical symptomatology, combined with its brevity and ease of use, makes it the tool of choice for assessing recovery in the Spanish healthcare system. Its implementation as a primary outcome measure in future clinical trials is recommended to exhaustively evaluate its sensitivity to change.

## Availability of Data and Materials

Not applicable.
